# Genetic Polymorphisms and Adverse Events on Unbound Imatinib and Its Active Metabolite Concentration in Patients With Gastrointestinal Stromal Tumors

**DOI:** 10.3389/fphar.2019.00854

**Published:** 2019-07-30

**Authors:** Yi Qian, Lu-Ning Sun, Yang-Jie Liu, Qiang Zhang, Jiang-Hao Xu, Zeng-Qing Ma, Xue-Hui Zhang, Hao Xu, Yong-Qing Wang

**Affiliations:** ^1^Research Division of Clinical Pharmacology, First Afﬁliated Hospital of Nanjing Medical University, Nanjing, China; ^2^General Surgery Department, First Afﬁliated Hospital of Nanjing Medical University, Nanjing, China; ^3^Department of Pharmacy, Jiangsu Shengze Hospital, Suzhou, China

**Keywords:** gastrointestinal, genetic polymorphism, adverse drug reactions, unbound drug concentration, protein concentration

## Abstract

Imatinib is a first-line drug for the treatment of gastrointestinal stromal tumors (GIST). This study aims to investigate the influence of different kinds of protein concentrations and genetic polymorphisms of metabolizing enzymes and drug transporters on unbound imatinib and its active metabolite N-desmethyl-imatinib concentration, as well as the relationship between adverse drug reactions (ADRs) and drug concentration. A total of 62 Chinese patients with GIST were genotyped for five single nucleotide polymorphisms (SNPs). Total and unbound 3h and trough concentration of imatinib and N-desmethyl-imatinib in GIST patients were determined by an LC-MS/MS method combined with an equilibrium dialysis. Single-Use Red Plate with inserts was used to separate the unbound drug. When the protein concentration became higher, the unbound imatinib and N-desmethyl-imatinib plasma concentration got higher (*p* < 0.05). Patients with GA genotype in *rs755828176* had significantly higher unbound N-desmethyl-imatinib dose-adjusted trough plasma concentrations (*p* = 0.012). Patients with CC genotype in *rs3814055* had significantly higher unbound imatinib dose-adjusted trough plasma concentrations (*p* = 0.040). The mean total imatinib C_3h_ of patients with ADRs (3.10 ± 0.96 µg/ml) was significantly higher than that of patients without ADRs (*p* = 0.023). The mean total N-desmethyl-imatinib C_3h_ of patients (0.64 ± 0.21 µg/ml) with ADRs was significantly higher than that of patients without ADRs (*p* = 0.004). The mean unbound N-desmethyl-imatinib C_3h_ of patients with ADRs (6.49 ± 2.53 ng/ml) was significantly higher than that of patients without ADRs (*p* = 0.042). The total and unbound C_3h_ of imatinib and N-desmethyl-imatinib in patients with ADRs was significantly higher than that in patients without ADRs (*p* < 0.05). Protein concentrations have great influence on the unbound imatinib and N-desmethyl-imatinib concentrations. The genetic polymorphisms of *CYP3A4 rs755828176* and *NR1I2 rs3814055* were significantly associated with unbound imatinib and N-desmethyl-imatinib dose-adjusted trough plasma levels. The total and unbound imatinib or N-desmethyl-imatinib concentration in patients with GIST was also significantly correlated with ADRs.

## Introduction

Imatinib (STI-571, Gleevec) is the first approved inhibitor of protein tyrosine kinases for the treatment of gastrointestinal stromal tumors (GIST) (Croom and Perry, 2003). Nearly 50% of patients with advanced GIST survived for more than 5 years because of the treatment with imatinib (Blanke et al., 2008; DeMatteo et al., 2009). Imatinib is approximately 95% bound to plasma proteins, mainly albumin and α1-acid glycoprotein (α1-AGP) (Widmer et al., 2006). The main metabolite of imatinib is CGP74588, also called N-desmethyl-imatinib, which shows comparable biologic activity regarding the BCR-ABL, PDGFR, and c-KIT tyrosine kinases to the parent imatinib (Delbaldo et al., 2006; van Erp et al., 2007). The plasma AUC for this metabolite is about 15% of the area under curve (AUC) for imatinib. The plasma protein binding of N-demethylated metabolite CGP74588 is similar to that of imatinib (Pharmaceuticals). A randomized phase II study in American showed that patients with imatinib trough concentration below 1100 ng/ml showed a lower rate of clinical benefit (Demetri et al., 2009). However, the specific imatinib trough concentration with which patients in China will achieve a good response has not been reported yet.

It is well-known that only the free drug is likely to equilibrate with the intracellular condition to exert its pharmacological action. The protein binding rates of imatinib and N-desmethyl-imatinib are over 95% so that a small change of protein binding rate may result in a significant impact on their free fraction and on their concentration–effect relationships. Therefore, determination of unbound plasma drug concentration is meaningful and necessary. Three main binding proteins in human plasma for imatinib and its metabolite are albumin (ALB), globulin (GLB), and α1-AGP. ALB is the most abundant protein in human plasma, accounting for 55% of the total plasma protein amount (Fasano et al., 2005). The concentration of GLB and α1-AGP is less than ALB in human plasma (Muller et al., 2017). Albumin is capable of binding a broad variety of drugs with sufficient affinity to impact on the pharmacologically active free fraction. α1-AGP also binds imatinib and its metabolite with higher affinity (Gambacorti-Passerini et al., 2013). Although the simultaneous determination of unbound imatinib and N-desmethyl-imatinib has recently been reported (Frank Streit et al., 2011; Arellano et al., 2012; Gandia et al., 2013), little is known about the relationship between these specific protein concentrations and unbound drug concentrations in GIST patients.

Imatinib is extensively metabolized by the cytochrome P450 enzyme system, and the main enzyme involved in imatinib metabolism *in vivo* is CYP3A4. Liu et al.’s (2017) study showed that subjects of *GG* genotype in *rs2242480* had significantly higher steady-state imatinib dose-adjusted trough plasma concentrations. In addition, it has been reported that imatinib is a substrate for the organic cation transporter 1 (OCT1, encoded by SLC22A1) and ATP-binding cassette subfamily G member 2 (ABCG2) (Nies et al., 2014; Skoglund et al., 2014; Koo et al., 2015). NR1I3 (Nuclear Receptor Subfamily 1 Group I Member 3, CAR for constitutive androstane receptor or constitutively activated receptor) is nuclear receptor, and its genetic polymorphisms have been reported to have influence on the metabolism and pharmacokinetics of drugs (Kurzawski et al., 2017; Mbatchi et al., 2018). NR1I2 (PXR for pregnane X receptor, or SXR for steroid and xenobiotic receptor) and NR1I3 are nuclear receptors that can affect the pharmacokinetics and therapeutic response to many drugs (Mbatchi et al., 2018). The CC genotype in *NR1I2 rs3814055* genetic variation was reported to have significantly higher steady-state imatinib dose-adjusted trough plasma concentrations (Liu et al., 2017). However, whether the single nucleotide polymorphisms (SNPs) are related to the unbound concentration of imatinib and N-desmethyl-imatinib or not is still unknown. Therefore, five SNPs (*CYP3A4 rs2242480*, *ABCG2 rs2231142*, *SLC22A1 rs755828176*, *NR1I3 rs2307418*, and *NR1I2 rs3814055*) were chosen in this article to investigate the influence of SNPs of metabolizing enzymes and transporters on the unbound imatinib and N-desmethyl-imatinib trough plasma concentrations.

In an international, randomized, open-label, phase 3 trial of imatinib, the most common adverse events were diarrhea, edema, fatigue, muscle spasm, nausea, and rash (Lipton et al., 2016). Another study showed that the trough level concentration was significantly higher in patients with thrombocytopenia compared with patients without the adverse event (P value 0.033) (Francis et al., 2015). However, the relationship between unbound imatinib and N-desmethyl-imatinib concentration and adverse drug reactions (ADRs) is still unknown. The threshold level of imatinib and N-desmethyl-imatinib for each adverse event also has not been reported yet.

This article aims to explore the association of plasma protein concentration with unbound imatinib or N-desmethyl-imatinib plasma concentration and the influence of SNPs on unbound imatinib or N-desmethyl-imatinib dose-adjusted trough plasma concentration, as well as the association of ADRs with unbound imatinib and N-desmethyl-imatinib concentration.

## Materials and Methods

### Study Design

This study was a single-center, observational phase IV trial conducted in the first affiliated hospital of Nanjing Medical University during December 2014 and September 2018. The clinical information, pathology examination, and genetic detection results were recorded in a case report form for each patient enrolled in our study. The study protocol was approved by the Ethics Committees of the first affiliated hospital of Nanjing Medical University (ethical approval code: 2013-SR-142). Our hospital was authenticated by AAHRPP (Association for Accreditation of Human Research Protection Programs, a non-profit organization in the USA). This study was also registered at the Chinese Clinical Trial Registry, and the registration number was ChiCTR-RNC-14004667.

### Patients

All patients have signed informed consents before entering this study. Blood samples were collected from 62 GIST patients during their clinical visits to the first affiliated hospital of Nanjing Medical University. Patients were recommended to receive imatinib mesylate (Gleevec^®^, Novartis Pharmaceuticals, Basel, Switzerland) at a dose of 400 mg daily. Of these 62 patients, 47 were treated with a dose of 400 mg/day, 12 were treated with 100–300 mg/day, and 3 were treated with 600 mg/day (**Table 1**). Patients were requested to come to our hospital for the imatinib plasma concentration detection at the first month and every 3 months after enrollment. All patients were at a steady state and treated with imatinib for at least 4 weeks. The blood collection time was before and 3 h after taking imatinib. Blood samples were centrifuged at 3,000 r/min, 4°C for 10 min, then separated and stored at −80°C for analysis.

**Table 1:** General information of 62 patients with GIST.

Characteristics	Patients with GIST	
Gender	Man	32
	Woman	30
Age (year)	Mean ± SD	54 ± 11.9
	Range	29–78
Body weight (kg)	Mean ± SD	61 ± 10.8
	Range	39–90
Body surface area (m^2^)	Mean ± SD	1.6 ± 0.2
	Range	1.3–2.1
ALB concentration (g/L)	Mean ± SD	43.9 ± 2.8
	Range	37.3–53.3
GLB concentration (g/L)	Mean ± SD	23.0 ± 3.5
	Range	15.6–27.9
Daily imatinib dose (mg)	100	1 (1.6%)
	200	2 (3.2%)
	300	9 (14.5%)
	400	47 (75.8%)
	600	3 (4.8%)

### Total and Unbound Drug Concentration Analysis

Liquid chromatography was performed on a Surveyor MS Pump Plus system (Thermo Fisher Scientific, USA). The mass spectrometric analysis was performed on a Finnigan TSQ Quantum Mass Spectrometer System (Thermo Fisher Scientific, USA). The separation was carried on a ZORBAX Eclipse Plus C18 column (2.1 mm × 50 mm, 1.8 μm, Agilent, USA). The mobile phase was consisted of 20 mM ammonium formate solution containing 0.1% formic acid–acetonitrile (75:25, v/v). The lower limit of imatinib and N-desmethyl-imatinib calibration curves in human plasma quantitation was 50 ng/ml, and the lower limit of the imatinib and N-desmethyl-imatinib calibration curve in PBS was 2 ng/ml. This method is accurate, reliable, and reproducible for the determination of total and unbound imatinib and N-desmethyl-imatinib plasma concentration. A mixed working solution containing imatinib and N-desmethyl-imatinib (100/100 μg/ml) was diluted with methanol to the concentration of 300 and 3,500 ng/ml. Albumin (ALB, 200 g/L) was diluted with PBS (phosphate-buffered saline, pH=7.20) to reach the concentration of 30, 45, and 60 g/L. Human α1-AGP was dissolved in PBS and then was diluted to 0.01, 0.10, and 1.00 g/L. Human globulin (GLB, 50 g/L) was diluted with PBS to reach the concentration of 10, 25, and 40 g/L. Mix protein solutions were prepared by mixing three proteins to reach the concentration of 40.01, 65.1, and 91 g/L. The spiked samples were prepared by mixing 200 μl protein solutions and 10 µL mixed IM/NDI working solutions. Single-Use Red Plate with inserts (Thermo Scientific, USA) was used to analyze the unbound drug concentration. The prepared sample was added into the sample chamber, and 350 μl PBS (phosphate-buffered saline, pH=7.2) was added into the buffer chamber. Pipette 50 or 100 μl volume was from both the plasma and buffer chambers after 2-h incubation. The calculation of plasma protein binding rate was according to Yukari Miyadera’s study (Yukari Miyadera et al., 2018).

### Genotyping Analysis

DNA was extracted from peripheral blood of 62 patients by using a Relaxgene Blood DNA System (TIANGEN, China) according to the manufacturers’ protocol. Genotyping for *CYP3A4 rs2242480*, *ABCG2 rs2231142*, *SLC22A1 rs755828176*, *NR1I3 rs2307418*, and *NR1I2 rs3814055* was carried out by polymerase chain reaction followed by Sanger sequencing. DNA sequencing was processed using BigDye^®^ Terminator Cycle Sequencing v3.1 kit and DNA Analyzer (3730xl, Applied Biosystems, USA), which was provided by Sangon Biotech (Shanghai, China).

### Evaluation of Adverse Drug Reactions

The information of ADRs was collected from patients’ case report forms. The ADRs were evaluated by the National Cancer Institute Common Terminology Criteria for Adverse Events version 4.0 (NCI CTCAE v4.0).

### Statistical Analysis

All data were performed on the SPSS program, version 22.0 (IBM, Chicago, IL, USA). The value of unbound imatinib and N-desmethyl-imatinib concentration was analyzed by one-sample Kolmogorov–Smirnov test. Imatinib and N-desmethyl-imatinib levels according to the genotypes of each SNP were compared using analysis of variance (ANOVA) and independent-samples T test. Imatinib and N-desmethyl-imatinib concentration of patients with or without ADRs were compared using ANOVA. A two-tailed *p* value <0.05 was considered statistically significant.

## Results

### Association of Different Protein Concentrations and Drug Concentrations

**Figure 1:**
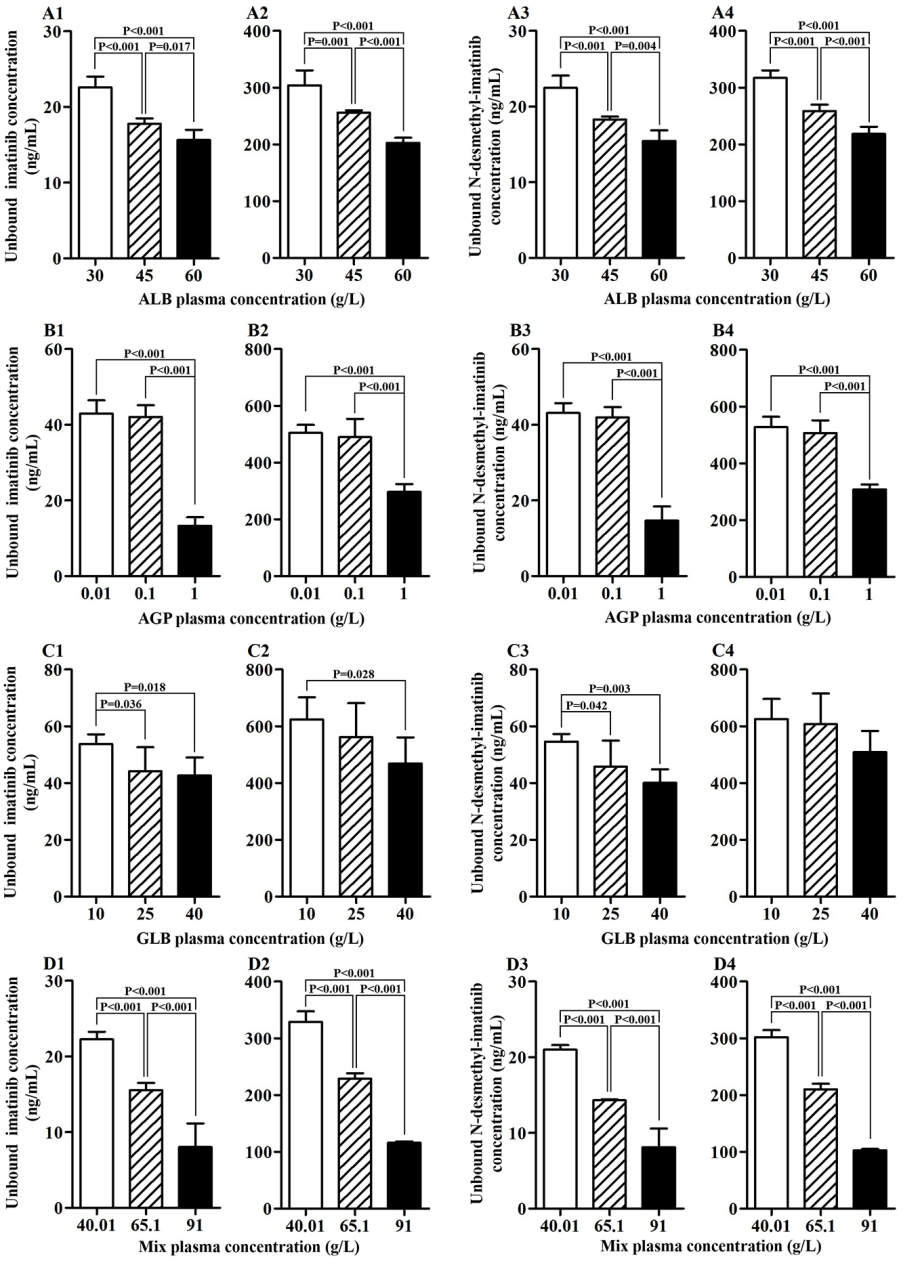
Association of protein concentration and unbound imatinib and N-desmethyl-imatinib concentration. **(A1**–**A4)** ALB plasma concentration vs unbound imatinib and N-desmethyl-imatinib concentration. **(B1**–**B4)** AGP plasma concentration vs unbound imatinib and N-desmethyl-imatinib concentration. **(C1**–**C4)** GLB plasma concentration vs unbound imatinib and N-desmethyl-imatinib concentration. **(D1**–**D4)** Mix plasma concentration vs unbound imatinib and N-desmethyl-imatinib concentration. **(A1**–**D1**, **A3**–**D3**: the total concentration of imatinib and N-desmethyl-imatinib is 300 ng/mL; **A2**–**D2**, **A4**–**D4**: the total concentration of imatinib and N-desmethyl-imatinib is 3,500 ng/mL).

Four kinds of protein solutions with different drug concentrations were prepared to examine the unbound concentration of imatinib and N-desmethyl-imatinib. As shown in **Figures 1A1–A4**, when the total drug concentration was the same, ALB concentration got larger and the unbound drug concentration became lower. Significant association between ALB concentration and unbound drug concentration was observed (*p* < 0.05). Moreover, when the ALB concentration was the same, the total drug concentration became larger and the unbound drug concentration got larger too. As shown in **Figures 1B1–B4**, significant differences between unbound drug concentration and AGP with the concentration of 0.01 or 0.10 g/L and 1.00 g/L were observed (*p* < 0.001). In addition, when the AGP concentration was the same (0.01, 0.10, and 1.00 g/L), the total drug concentration got larger and the unbound drug concentration got larger too. As shown in **Figures 1C1–C4**, when the GLB concentration got larger, the unbound drug concentration became lower. Statistical variations between GLB with the concentration of 10 and 40 g/L in unbound drug concentration were observed (*p* < 0.05) at the same drug concentration. Moreover, when the GLB concentration was same, the total drug concentration got larger and the unbound drug concentration got larger too. Significant differences between GLB concentration and unbound drug concentration were observed (*p* < 0.001) (**Figures 1D1–D4**). Meanwhile, the total drug concentration got larger and the unbound drug concentration got larger too.

### The Total and Unbound Imatinib and N-Desmethyl-Imatinib Concentration of Patients

**Figure 2:**
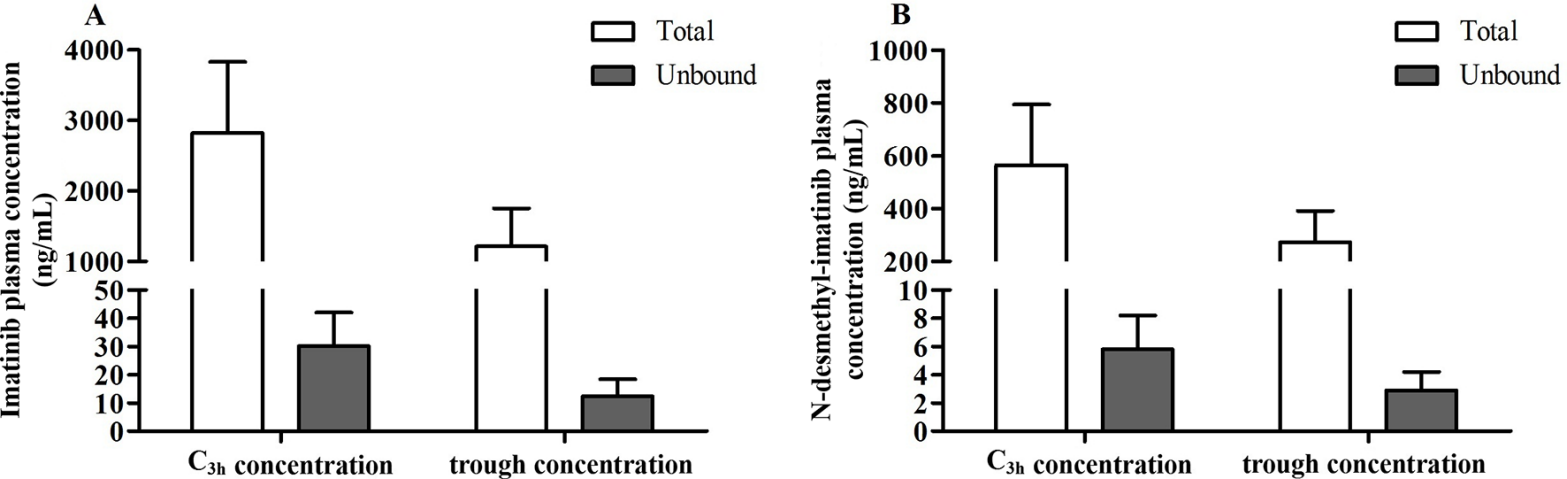
Association of total and unbound imatinib **(A)** and N-desmethyl-imatinib **(B)** concentration.

**Figure 3:**
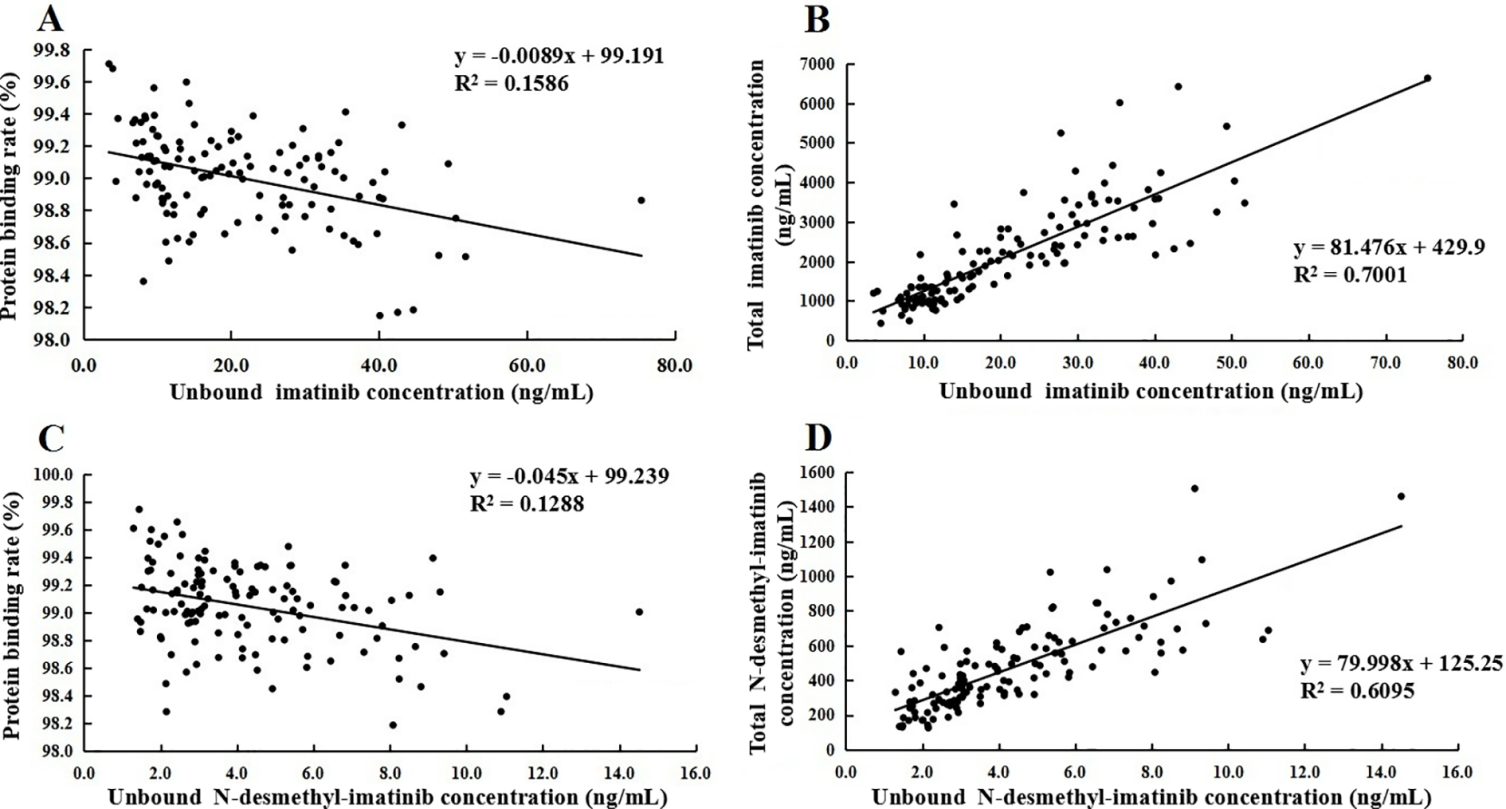
**(A)** Association of unbound imatinib concentration and plasma protein binding rate. **(B)** Association of unbound N-desmethyl-imatinib concentration and plasma protein binding rate. **(C)** Association of total imatinib concentration and unbound imatinib concentration. **(D)** Association of total N-desmethyl-imatinib concentration and unbound N-desmethyl-imatinib concentration.

The protein binding rate of imatinib ranged from 98.1% to 99.7%, while that of N-desmethyl-imatinib was 98.2% to 99.7%. The total and unbound imatinib and N-desmethyl-imatinib concentration was shown in **Figure 2**. The mean total C_3h_ of imatinib and N-desmethyl-imatinib in 62 patients with GIST was (2,821.8 ± 1,008.0) and (565.1 ± 228.7) ng/ml, while the unbound C_3h_ was (30.1±12.0) and (5.8±2.4) ng/ml. The mean total trough concentration of imatinib and N-desmethyl-imatinib in 62 patients with GIST was (1,212.3±541.3) and (272.1±119.5) ng/ml, while the unbound trough concentration was (12.3±6.1) and (2.9±1.3) ng/ml. As shown in **Figure 3**, when the unbound imatinib and N-desmethyl-imatinib concentration became higher, the protein binding rate got lower and the total imatinib and N-desmethyl-imatinib concentration got larger, which was in accord with our experiments *in vitro*.

### Association of SNPs With Unbound Dose-Adjusted Plasma Concentration of Imatinib and N-Desmethyl-Imatinib

**Figure 4:**
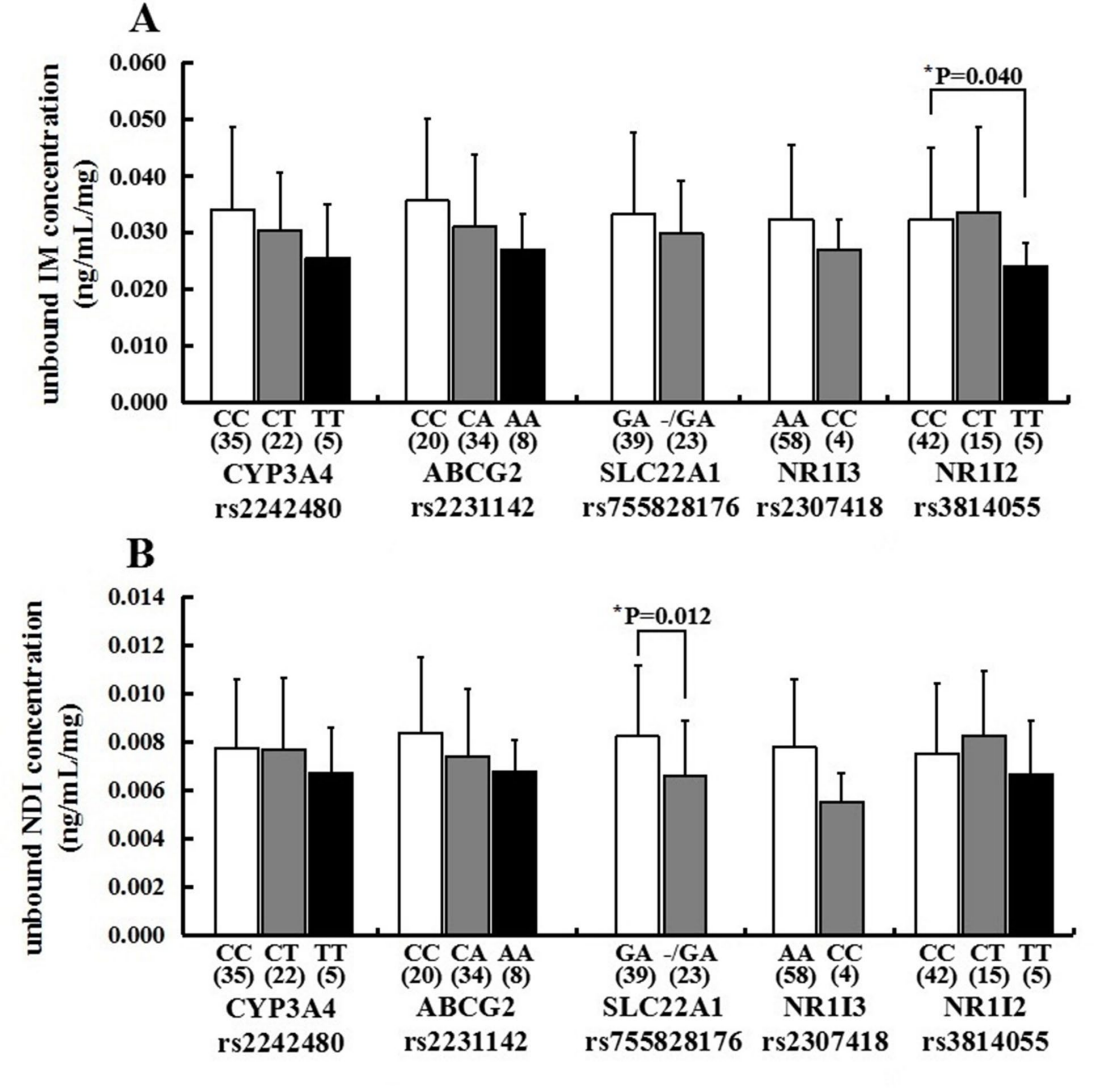
Association of *CYP3A4 (rs2242480)*, *ABCG2 (rs2231142)*, *SLC22A1 (rs755828176)*, *NR1I3 (rs2307418)*, and *NR1I2 (rs3814055)* genotypes with the unbound imatinib **(A)** and N-desmethyl-imatinib **(B)** dose-adjusted trough plasma concentrations in 62 Chinese GIST patients (*p < 0.05: *SLC22A1 rs755828176* GA vs -/GA, mean unbound N-desmethyl-imatinib dose-adjusted trough concentration; *NR1I2 rs3814055* CC vs TT, mean unbound imatinib dose-adjusted rough concentration).

For *CYP3A4 rs2242480*, there is no significant difference in the mean unbound imatinib and N-desmethyl-imatinib dose-adjusted trough plasma concentration in observed genotypes (*p* > 0.05) (**Figure 4**). For *ABCG2 rs2231142*, there is no significant difference in the mean unbound imatinib and N-desmethyl-imatinib dose-adjusted trough plasma concentration in observed genotypes (*p* > 0.05) (**Figure 4**). For *NR1I3 rs2307418*, there is no significant difference in the mean unbound imatinib and N-desmethyl-imatinib dose-adjusted trough plasma concentration in observed genotypes (*p* > 0.05) (**Figure 4**). Marked variability in the dose-adjusted unbound trough levels of imatinib and N-desmethyl-imatinib was seen in patients with different genotypes. For *SLC22A1 rs755828176*, the unbound N-desmethyl-imatinib dose-adjusted trough concentration of patients with GA genotype (0.0082 ± 0.0029 ng/ml/mg) was significantly higher than that in patients with -/GA genotype (0.0066±0.0023 ng/ml/mg) (*p* = 0.012). For *NR1I2 rs3814055*, the unbound imatinib dose-adjusted trough concentration of patients with CC genotype (0.0324 ± 0.0127 ng/ml/mg) was significantly higher than that in patients with TT genotype (0.0241±0.0042 ng/ml/mg) (*p*=0.040) (**Figure 4**).

### Association of Total and Unbound Drug Concentration With ADRs

**Figure 5:**
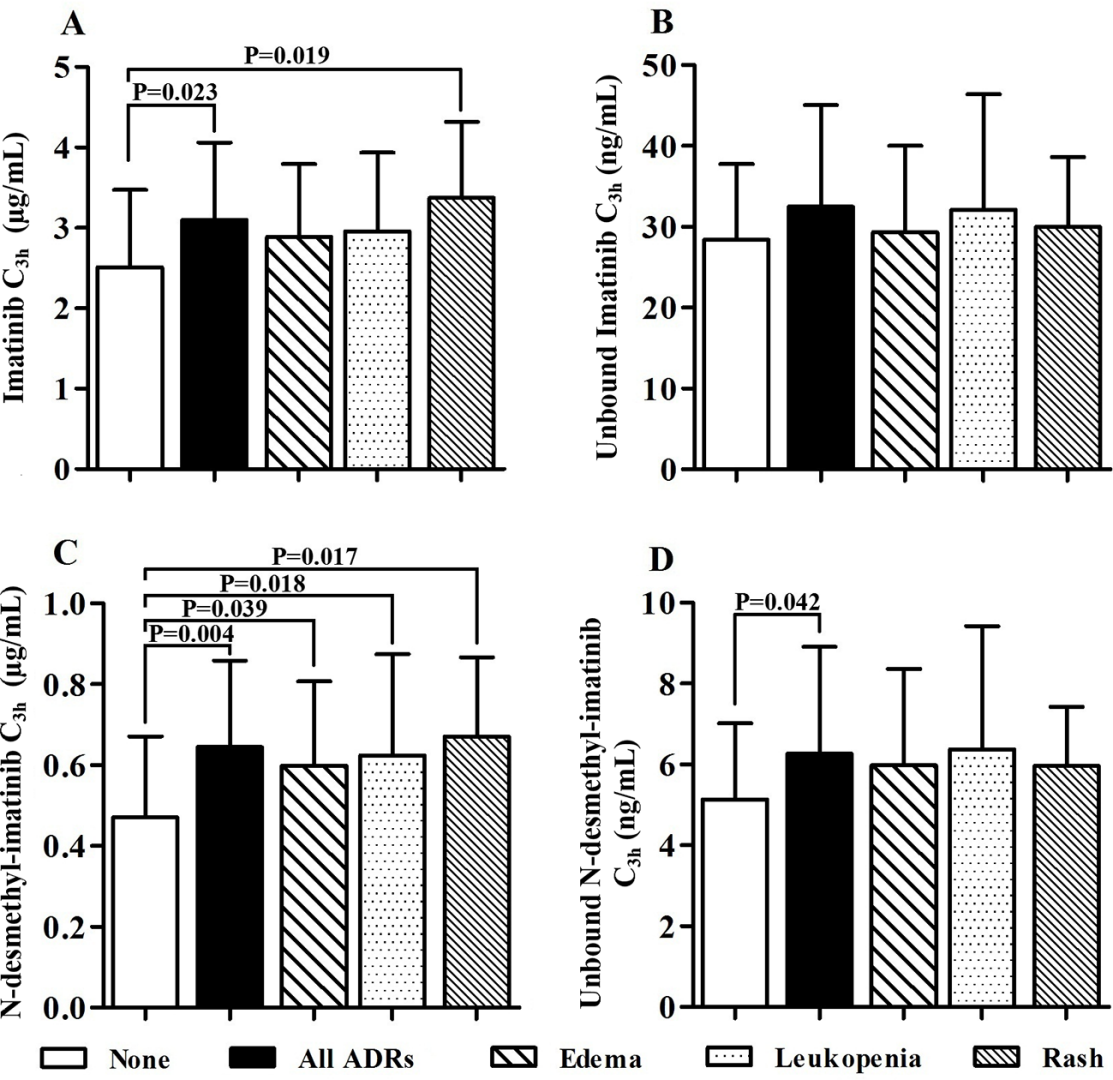
Association of total and unbound drug concentration and adverse drug reactions (ADRs) (**A**: The imatinib C_3h_ of patients without ADRs was significantly lower than that of patients with any ADRs and patients with rash. **B**: There was no statistical difference between the unbound imatinib C3h of patients with and without ADRs. **C**: The N-desmethyl-imatinib C_3h_ of patients without ADRs was significantly lower than other groups. **D**: The unbound N-desmethyl-imatinib C_3h_ of patients without ADRs was significantly lower than that of patients with any ADRs).

The most frequently ADRs occurred in 62 GIST patients were edema, leukopenia, and rash. Moreover, the majority of ADRs were grade 1–2. We found one patient with grade 3 leukopenia and one patient with grade 3 edema and rash. The mean total and unbound C_3h_ of imatinib and its metabolite N-desmethyl-imatinib are presented in **Table 2**. The mean total imatinib C_3h_ of patients with ADRs (3.10 ± 0.96 µg/ml) was significantly higher than that of patients without ADRs (*p* = 0.023), and the mean total imatinib C_3h_ of patients with rash (3.37 ± 0.95 µg/ml) was also significantly higher than that of patients without ADRs (*p* = 0.019) (**Figure 5**). The mean total N-desmethyl-imatinib C_3h_ of patients with any kinds of ADRs was significantly higher than that of patients without ADRs. The mean unbound N-desmethyl-imatinib C_3h_ of patients with ADRs (6.49 ± 2.53 ng/ml) was significantly higher than that of patients without ADRs (*p* = 0.042).

**Table 2:** Adverse drug reactions (ADRs) of 62 GIST patients.

ADRs	Number of patients (%)	Total C_3h_ (ﴽµg/mL±SD)		Unbound C_3h_ (ng/mL±SD)	
		Imatinib	N-desmethyl-imatinib	Imatinib	N-desmethyl-imatinib
None	22 (35.5)	2.51 ± 0.96	0.47 ± 0.20	28.39 ± 9.37	5.14 ± 1.88
All ADRs	40 (64.5)	3.10 ± 0.96*	0.64 ± 0.21*	32.48 ± 12.57	6.49 ± 2.53*
Edema	29 (46.8)	2.89 ± 0.90	0.60 ± 0.21*	29.30 ± 10.70	5.98 ± 2.38
Leukopenia	25 (40.3)	2.96 ± 0.98	0.62 ± 0.25*	32.10 ± 14.28	6.37 ± 3.05
Rash	13 (21.0)	3.37 ± 0.95*	0.67 ± 0.20*	29.98 ± 8.66	5.96 ± 1.47

*p < 0.05: None vs All ADRs, total imatinib and N-desmethyl-imatinib C_3h_, unbound N-desmethyl-imatinib C_3h_; None vs Edema, total N-desmethyl-imatinib C_3h_; None vs Leukopenia, total N-desmethyl-imatinib C_3h_; None vs Rash, total imatinib and N-desmethyl-imatinib C_3h_.

## Discussion

At present, there are several methods for determining unbound imatinib and N-desmethyl-imatinib plasma concentrations such as equilibrium dialysis (Mano and Kusano, 2015), ultrafiltration (Tang et al., 2006; Arellano et al., 2012; Gandia et al., 2013), and ultracentrifugation (Tokui et al., 2004). Ultrafiltration and ultracentrifugation have been commonly used to determine the unbound concentrations. However, one of the major disadvantages of ultrafiltration is the protein leakage, and ultracentrifugation also has the disadvantage of expensive equipment and physical phenomena such as sedimentation, reverse diffusion, and viscosity, which may affect the accurate quantification of unbound drug concentration. The rapid equilibrium dialysis method that we used is easy to operate and can reduce the drug adsorption on the surface of the equilibrium dialysis equipment. The balance time is shortened to about 2 h, and less volume of samples is needed.

It is widely accepted that the efficacy of a drug is related to the exposure of the unbound drug concentration rather than total drug concentration. This study showed that higher concentration of proteins could result in significant reduction of unbound imatinib and N-desmethyl-imatinib concentration and then affect the pharmacological action. We consider that the protein concentration should be taken into account during imatinib treatment of GIST patients.

Patients were recommended to receive imatinib mesylate (GLEEVEC^Ⓡ^, Novartis Pharmaceuticals, 2001) at a dose of 400 mg/day. Moreover, patients with disease progression or postoperative recurrence were given the recommendation of increasing the original dose to 600 mg/day. If intolerant to imatinib or suffered from serious adverse events, patients were recommended to reduce the dose to 100–300 mg/day.

There are several reports about the relationship between imatinib trough plasma concentration and metabolizing enzymes or transporters. However, the influence of metabolizing enzymes and transporters SNPs on unbound imatinib and N-desmethyl-imatinib trough plasma concentrations has not been reported yet. In this study, we found that there were no significant differences in the mean unbound imatinib and N-desmethyl-imatinib dose-adjusted plasma concentrations of patients with *CYP3A4 (rs2242480)*, *ABCG2 (rs2231142)*, and *NR1I3 (rs2307418)*. However, a significant difference was observed in unbound N-desmethyl-imatinib dose-adjusted trough plasma concentrations of patients with *SLC22A1 (rs755828176)* genotypes, and the unbound N-desmethyl-imatinib dose-adjusted trough plasma concentrations in GA carriers were significantly higher than that in mutant types (-/GA) (*p* = 0.012), which means that patients with GA genotypes of *rs755828176* may need a lower dose therapy. Another significant difference was observed in the mean unbound imatinib dose-adjusted plasma concentrations of patients with *NR1I2 (rs3814055)* genotypes. The unbound imatinib dose-adjusted trough concentration of patients with CC genotype was significantly higher than that in patients with TT genotype (*p* = 0.040). It was reported that CC genotype in *rs3814055* had significantly higher steady-state imatinib dose-adjusted trough plasma concentrations, which was in agreement with the results of our study (Liu et al., 2017). These two SNPs may be able to predict the effect of imatinib therapy. Determination of unbound drug concentration and genotyping of *SLC22A1 (rs755828176)* and *NR1I2 (rs3814055)* genes may be considered in patients with GIST to individualize the therapy and optimize the clinical outcomes. However, the mechanism of how *SLC22A1 (rs755828176)* and *NR1I2 (rs3814055)* SNPs influence imatinib and N-desmethyl-imatinib plasma concentration has not been clearly reported yet. Further research is needed to confirm our findings.

Our study showed that ADRs including edema, leukopenia, and rash were significantly related to the total N-desmethyl-imatinib plasma concentrations in patients with GIST, which indicates that if the total or unbound drug concentration reaches the threshold of imatinib or N-desmethyl-imatinib, the patients may have greater probability of ADRs like edema, leukopenia, and rash. Therefore, it is important to determine not only the total drug concentration but also the unbound imatinib and N-desmethyl-imatinib concentration to prevent ADRs in GIST patients.

## Conclusion

Protein concentrations have great influence on unbound imatinib and N-desmethyl-imatinib concentration. The protein concentration should be taken into account during imatinib treatment of GIST patients. *SLC22A1 (rs755828176)* and *NR1I2 (rs3814055)* SNPs may be able to predict the effect of imatinib therapy in Chinese GIST patients. Mean imatinib and N-desmethyl-imatinib C_3h_ of GIST patients with edema, leukopenia, or rash was significantly higher than that of patients without ADRs.

## Data Availability

The raw data supporting the conclusions of this manuscript will be made available by the authors, without undue reservation, to any qualified researcher.

## Ethics Statement

This study was carried out in accordance with the recommendations of “Good Clinical Practice Guideline, China Food and Drug Administration” with written informed consent from all subjects. All subjects gave written informed consent in accordance with the Declaration of Helsinki. The protocol was approved by the “The Ethics Committee of First Affiliated Hospital with Nanjing Medical University.” This study was registered at the Chinese Clinical Trial Registry and the registration number was ChiCTR-RNC-14004667. The study protocol was approved by the Ethics Committees of the First Affiliated Hospital of Nanjing Medical University (ethical approval code: 2013-SR-142). Our hospital was authenticated by AAHRPP (Association for Accreditation of Human Research Protection Programs, a non-profit organization in the USA).

## Author Contributions

L-NS, X-HZ, HX, and Y-QW contributed to the conception and design of the study. YQ and QZ performed the statistical analysis. YQ wrote the first draft of the manuscript. YQ, Y-JL, QZ, J-HX, and Z-QM wrote sections of the manuscript. All authors contributed to manuscript revision and read and approved the submitted version.

## Funding

This project was funded by the grants from the National Natural Sciences Foundation of China, grant numbers 81503160, 81673515, and 81870436; Natural Science Foundation of Jiangsu Province, grant number BK20161591; Jiangsu Provincial Medical Youth Talent, grant number QNRC2016215; Suzhou science and education Youth Project, grant number KJXW2016067; and Suzhou industrial technology innovation, grant number SYSD2016046.

## Conflict of Interest Statement

The authors declare that the research was conducted in the absence of any commercial or financial relationships that could be construed as a potential conflict of interest.
