# Supplementary material for: Genetic Polymorphisms and Adverse Events on Unbound Imatinib and Its Active Metabolite Concentration in Patients With Gastrointestinal Stromal Tumors
Source: Front Pharmacol. 2019 Jul 30;10:854. doi: 10.3389/fphar.2019.00854 (PMC6682687; doi:10.3389/fphar.2019.00854)
Supplement: Supplementary file 1 [file Table_1.docx]

Table 1 Schedule of Study Drug Administration and Data Collection

| Items | Second week after operation | Fourth week after operation | 0.5, 1, 2, 3, 6 months after administration | 12, 18, 24 months after administration |
| --- | --- | --- | --- | --- |
| Selection/exclusion criteria | √ |  |  |  |
| Sign informed consent | √ |  |  |  |
| Fill in patient information form | √ |  |  |  |
| Examination of liver/kidney function and electrolytes | √ |  |  |  |
| Blood routine examination | √ |  |  |  |
| Start taking imatinib 400 mg/day |  | √ |  |  |
| Determination of imatinib plasma concentration |  |  | √ | √ |
| Examination of liver/kidney function and electrolytes |  |  | √ | √ |
| Blood routine examination |  |  | √ | √ |
| Recording adverse events |  |  | √ | √ |
